# Direct Oral Anticoagulant Treatment for Atherosclerosis-Induced Aortic Mural Thrombus in an Elderly Male With Aspirin Resistance

**DOI:** 10.7759/cureus.62691

**Published:** 2024-06-19

**Authors:** Ryuta Sugihara, Hidetaka Kioka, Yasushi Sakata

**Affiliations:** 1 Department of Cardiovascular Medicine, Osaka University Graduate School of Medicine, Osaka, JPN

**Keywords:** aspirin resistance, atherosclerosis, non-progressive, direct oral anticoagulant therapy, aortic mural thrombosis

## Abstract

Aortic mural thrombus is associated with atherosclerosis in a vast majority of cases and could result in multiple organ damage, leading to higher morbidity and mortality rates. Although aspirin could be effective for primary prevention in atherosclerosis-induced aortic mural thrombus, aspirin resistance, which refers to the inadequate response to aspirin therapy, allows the progression of thrombus. Classically, warfarin could be an effective treatment for thromboembolic diseases, while in recent years, direct oral anticoagulants (DOACs) have shown superior safety and efficacy, particularly in elderly patients. This report presents the case of an elderly male with chronic aortic mural thrombi due to atherosclerosis and aspirin resistance who achieved favorable outcomes following treatment with DOACs. DOACs could be a possible option for managing aortic mural thrombus with aspirin resistance.

## Introduction

Aortic atherosclerotic mural thrombus is a frequent accidental finding on imaging examinations. This disease is correlated with coronary vascular diseases in retrospective studies, but whether it is an independent predictor remains unconfirmed [1,2]. It commonly causes distal organ and acute limb ischemia, leading to increased morbidity and mortality. The optimal management is still controversial according to current guidelines [3], although some guidelines recommend the use of aspirin in patients with asymptomatic peripheral artery disease [3,4]. Altogether, the presence of aortic plaques with mural thrombus in an asymptomatic patient does not, in itself, mandate antithrombotic therapy, but it should lower the threshold for initiating medication for primary prevention of thromboembolism.

Aspirin resistance may be caused by an increased sensitivity of platelets to collagen and affects 25% of patients with vascular disease, which increases their risk of experiencing major adverse limb and cardiovascular events, including aortic atherosclerotic mural thrombus [5,6]. Currently, there is no clear alternative to aspirin for patients who are resistant to its effects.

Anticoagulant agents have been used for primary or secondary prophylaxis and reduced vascular events in patients with thromboembolic aortic disease compared to antiplatelet therapy [7]. However, various studies have indicated that direct oral anticoagulants (DOACs) could be a strong candidate due to their ability to significantly reduce platelet aggregation [8] and decrease the incidence of adverse cardiovascular events [9,10]. Furthermore, DOACs have demonstrated efficacy and safety compared to warfarin, particularly in patients with atrial fibrillation [11] and/or chronic coronary artery disease [9,12].

Considering these findings and insights, DOACs could be a useful, feasible, and preferable option for the treatment of aortic atherosclerotic thrombus in elderly patients with aspirin resistance. However, there is currently no literature available that has specifically examined the efficacy of this approach. This case demonstrates that DOACs could be a safe and effective treatment option, as shown by our favorable results with DOAC treatment and the absence of bleeding events.

## Case presentation

An 80-year-old male patient was referred to our hospital with asymptomatic aortic mural thrombi in descending aorta. His medical history included coronary artery disease, diabetes mellitus, hypertension, and dyslipidemia. The patient had undergone percutaneous coronary intervention eight years previously and had drug-eluting stents implanted in both the left anterior descending and right coronary arteries. Following the procedure, the administration of clopidogrel (75 mg/day) was stopped one year after the stent placement, while aspirin therapy (100 mg/day) was continued.

**Figure 1:**
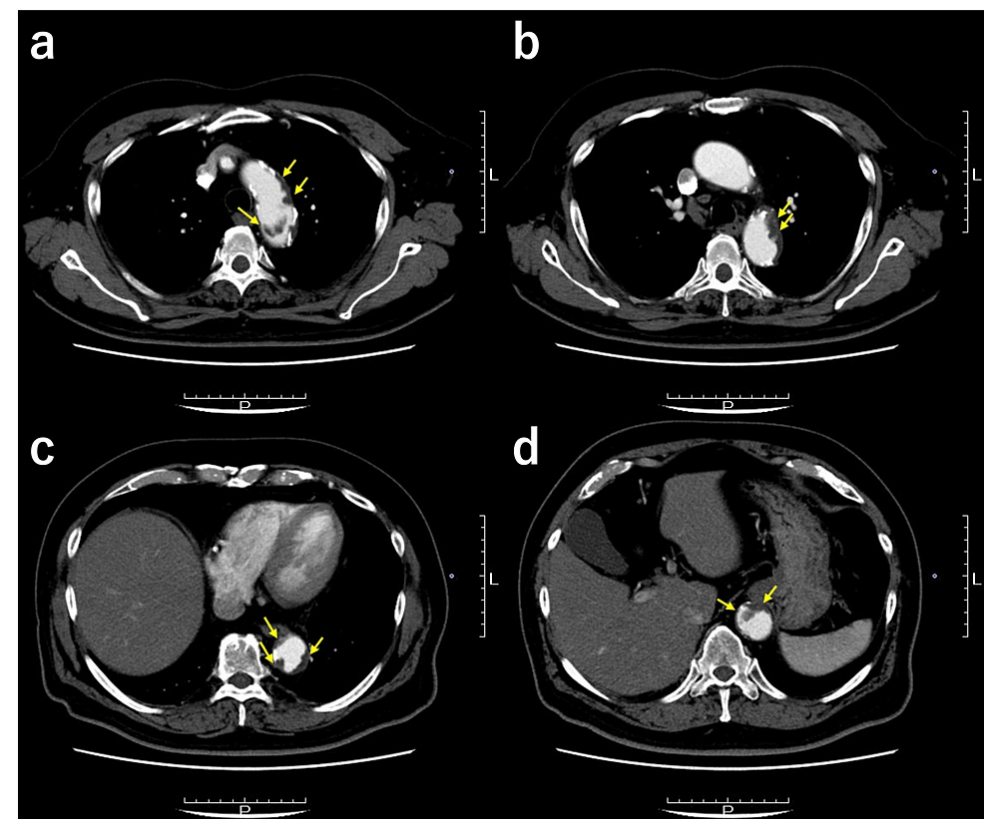
Computer tomography angiography images of a giant mural thrombus from the aortic arch (a, b) to the descending aorta (c, d), indicated in yellow arrows.

Computer tomography (CT) angiography revealed massive mural thrombi from the aortic arch to the descending aorta (Figure 1), prompting further investigation into why the thrombus had developed despite the patient's daily aspirin regimen. Blood examinations did not indicate any underlying hypercoagulable or fibrinolytic disorders (Table 1). 

**Table 1:** Laboratory examination before the treatment of direct oral anticoagulants.

Parameters	Results	Reference ranges
Biochemical test		
Serum creatinine (mg/dL)	0.81	0.60–1.10
Urea (mg/dL)	14	8.0–22.0
Glucose (mg/dL)	113	80–113
Hemoglobin A1c (%)	7.3	4.6–6.2
Albumin (g/dL)	3.9	3.8–5.0
Total bilirubin (mg/dL)	0.9	0.3–1.2
Alanine transaminase (U/L)	27	8–42
Aspartate transaminase (U/L)	30	13–33
Alkaline phosphatase (U/L)	72	115–359
Choline esterase (U/L)	341	214–466
Total cholesterol (mg/dL)	155	128–219
High-density lipoprotein cholesterol (mg/dL)	39	40–96
Low-density lipoprotein cholesterol (mg/dL)	67	70–140
Hematological test		
White blood cells (×10^3^/μL)	6.45	4.0–9.0
Hemoglobin (g/dL)	14.6	13.5–18.0
Platelets (×10^3^/μL)	187	150–350
Prothrombin time-international normalized ratio (-)	1.04	0.90–1.14
Activated partial thromboplastin time (sec)	28	24–40
Fibrinogen (mg/dL)	298	150–340
Anti-thrombin III (%)	85	80–120
plasminogen activity (%)	96	80–130
D-dimer (μg/mL)	0.7	<1.0
Fibrinogen/fibrin degradation products (μg/mL)	2.2	<5.0
Thrombin-antithrombin complex (ng/mL)	1.4	<4.0
Protein C activity (%)	95	62–131
Protein S activity (%)	89	64–149
Tumor-associated antigen test		
Carbohydrate antigen 19-9 (U/mL)	11.6	<37.0
Carcinoembryonic antigen (ng/mL)	2.0	0.1–5.0
Squamous cell carcinoma antigen (ng/mL)	1.0	<1.5
Alpha-fetoprotein (ng/mL)	3.0	<10
Prostate-specific antigen (ng/mL)	0.4	0–4.0
Protein induced by vitamin K absence or antagonist II (mAU/mL)	25	<40
Autoimmune-related test		
Lupus anticoagulant (-)	1.09	<1.2
Antinuclear antibody (titer)	< 40	<40
Serine proteinase 3-antineutrophil cytoplasmic antibody (U/mL)	1.0	<2.0
Myeloperoxidaseantineutrophil cytoplasmic antibody (U/mL)	1.0	<3.5
Anti-cardiolipin antibody, IgG (U/mL)	6.9	<12.3
Anti-cardiolipin antibody, IgM (U/mL)	1.0	<20.8

**Figure 2:**
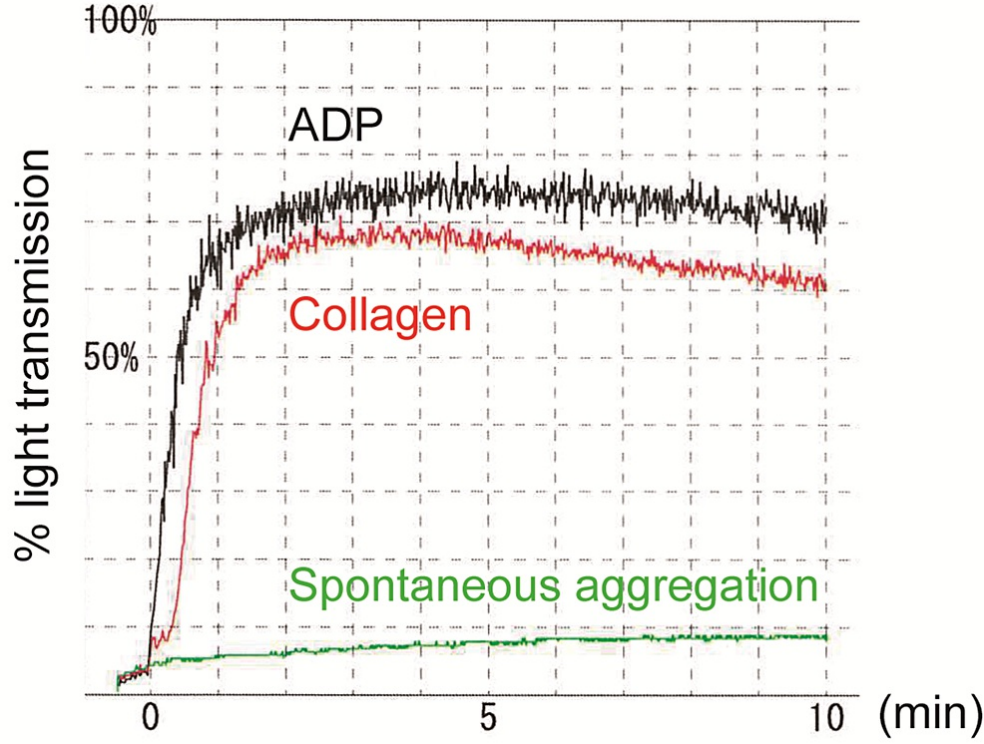
Platelet aggregation curves illustrating the responses to adenosine diphosphate (ADP, black) at 10 μM, collagen (red) at 2.0 μg/ml, and spontaneous aggregation (green) in this patient. In typical cases, the collagen-induced platelet aggregation induced by collagen should exhibit complete suppression, while the ADP-induced platelet aggregation should demonstrate slight suppression curve. However, in this patient, no inhibition was observed for either of these stimuli.

Aspirin is a drug that inhibits collagen-induced aggregation, which is associated with primary aggregation in the platelet aggregation process, while clopidogrel and ticlopidine are drugs that inhibit ADP-induced aggregation, which is associated with secondary aggregation [13]. In the platelet aggregation test, it was expected that collagen-induced platelet aggregation would be completely inhibited by aspirin. However, in this case, aspirin was found to have a low inhibitory effect with the collagen-induced platelet-rich plasma aggregation (70% of maximal aggregation, respectively), leading us to consider it as aspirin resistance (Figure 2). Furthermore, small thrombi had been detected in a previous examination six years earlier, suggesting that the thrombi had gradually progressed over time. Thus, we diagnosed the patient with aortic atherosclerotic mural thrombus with aspirin resistance.

**Figure 3:**
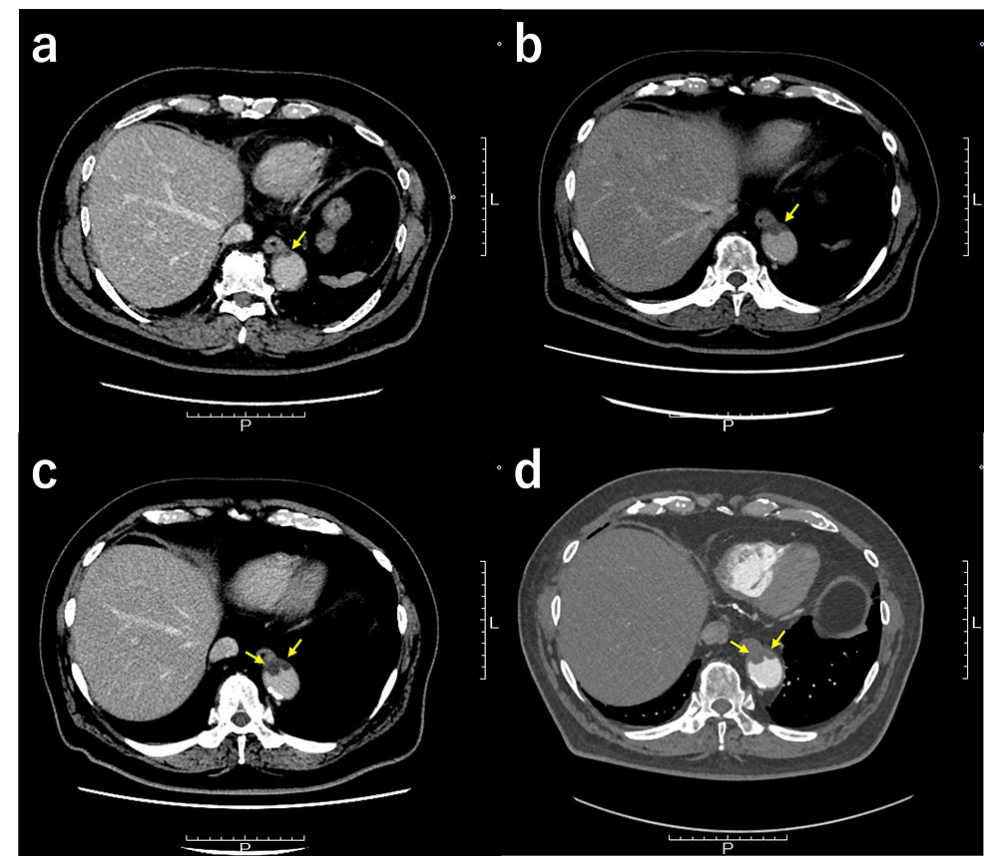
Comparison of the effects of aspirin and direct oral anticoagulant (DOAC) on the progression of thrombosis over a period of six months, indicated in yellow arrows. (a) Initial detection of the aortic mural thrombus. (b) Lack of thrombus progression prevention with aspirin treatment over a six-month duration. (c) Baseline condition prior to the administration of DOAC. (d) Effective inhibition of thrombus progression with DOAC treatment over the corresponding six-month period.

Considering his age, absence of symptoms, and no signs of organ damage in examinations, we opted for a conservative therapeutic strategy. Specifically, we switched the patient's aspirin therapy to DOAC, edoxaban at a dosage of 60 mg daily. Over the course of six months, the patient did not experience any bleeding or thrombotic events. Follow-up CT angiography demonstrated that the DOAC was highly effective in halting the progression of the thrombus, as compared to the six-month duration of aspirin therapy (Figure 3). Currently, the patient remains in good condition and is undergoing regular follow-up every three months at our hospital.

## Discussion

The mechanism of aortic atherosclerosis is similar to that of coronary atherosclerosis. The pathologic characteristics of aortic atherosclerosis have been described extensively in postmortem studies. This atherosclerosis begins with intimal thickening due to smooth muscle cell accumulation, followed by lipid entrapment and foam cell formation. Continued lipid deposition leads to a necrotic core of lipid and cell debris. Atherosclerotic lesions show varying fibrosis, calcification, and inflammation, with advanced plaques often exhibiting surface ulceration and thrombus attachment [14]. Diagnosis of aortic atherosclerosis utilizes various imaging techniques, transoesophageal echocardiography (TEE), contrast-enhanced CT angiography, and magnetic resonance angiography (MRA). TEE invasively provides reliable information on plaque mobility, ulceration, thickness, and composition. CT angiography and MRA can non-invasively provide detailed information about plaque distribution, morphology, and composition. These examinations are preferred for imaging the aorta and its branches, although both techniques have limitations, such as the inability to identify mobile components within plaques. Primary aortic mural thrombus, which occurs in the absence of atherosclerosis, is associated in the majority of cases with abnormalities of coagulation function, such as antiphospholipid syndrome, underlying malignancy, and autoimmune disorders [2]. In this patient, the diagnosis of aortic atherosclerotic mural thrombosis with calcifications was based on the patient's prophylactic use of aspirin, absence of thromboembolism history or abnormalities in blood tests, and the presence of coexisting diabetes mellitus and coronary arteriosclerosis, although no pathological examination or transoesophageal echocardiography was performed.

Aspirin is an integral component in the secondary prevention of cardiovascular disease by inhibiting the irreversible activity of platelet cyclooxygenase-1, which prevents the production of the potent pro-aggregatory thromboxane A_2_. Clinical factors, particularly poor adherence to medication, could be considered as potential causes of aspirin resistance [5,13]. Based on the information obtained from the interview, it appears that this patient demonstrated good adherence to medication, although this assessment is subjective and has not been objectively confirmed or validated. Therefore, a platelet aggregation test should be performed to determine a failure of aspirin to evaluate platelet function [13].

Furthermore, accelerated platelet turnover due to inflammatory processes such as atherosclerosis or comorbidities can lead to faster platelet regeneration, which may result in aspirin resistance and atherosclerosis [13]. Chronic arterial inflammation associated with long-standing diabetes mellitus and coronary artery disease may have led to aspirin resistance in our patient. Furthermore, several studies have reported that DOACs have anti-inflammatory effects [15]. This suggests that DOACs might have the potential to inhibit thrombus formation caused by chronic inflammation, which could also contribute to efficacy in cases of aspirin resistance.

Thrombolytic and antithrombotic therapies could be effective treatment options for aortic mural thrombus, although their use carries a risk of bleeding events [3-5, 7]. In our case, the patient’s age was high, and the aortic mural thrombus progressed over several years without any noticeable symptoms, which limits the potential effectiveness of these therapies. Therefore, we prioritized halting the progression of the disease using DOACs while minimizing the risk of bleeding events.

## Conclusions

To the best of our knowledge, this is the first reported successful treatment of DOACs instead of warfarin in aortic atherosclerotic mural thrombus with aspirin resistance, resulting in cessation of disease progression without any adverse bleeding events. The patient was on prophylactic aspirin to prevent coronary artery occlusion, and he had no history of venous thromboembolic disease in the past, yet he developed this aortic mural thrombus. Thrombi embolism can be critical events such as acute limb ischemia and renal infarction. Therefore, patients with other risk factors and comorbidities should be followed up closely with serial physical examinations and the platelet aggregation test. Complaints made by patients on aspirin with high risk should be taken seriously and appropriate tests should be considered according to the complaint. In addition, anticoagulants could be a safe and favorable treatment for aortic atherosclerotic mural thrombus. Further studies are warranted to confirm these findings and determine the optimal management approach for this challenging condition.
